# A Vegan Diet Epigenetically Modulates Inflammatory Pathways and Biological Aging: Genome‐Wide DNA Methylation Analysis of a One‐Month Isocaloric Vegan Versus Meat‐Rich Dietary Intervention

**DOI:** 10.1002/mco2.70899

**Published:** 2026-08-03

**Authors:** L. Karbacher, Jerome Mertens, S. Kowarschik, A. K. Lederer, M. Ku, R. Huber, Maximilian Andreas Storz

**Affiliations:** ^1^ Department of Neurosciences University of California San Diego La Jolla California USA; ^2^ Laboratory of Genetics The Salk Institute for Biological Studies La Jolla California USA; ^3^ Department of Internal Medicine II Center for Complementary Medicine Medical Center–University of Freiburg, Faculty of Medicine, University of Freiburg Freiburg Germany; ^4^ Department of General Visceral and Transplantation Surgery University Medical Center Mainz Mainz Germany; ^5^ Division of Pediatric Hematology and Oncology Department of Pediatrics and Adolescent Medicine Faculty of Medicine Medical Center‐University of Freiburg Freiburg Germany

**Keywords:** differential DNA methylation, epigenetic DNA methylation age analysis, epigenetics, genome‐wide DNA methylome, nutrition, vegan diet

## Abstract

We explored the epigenomic effects of vegan diet (VD) versus meat‐rich diet (MR) and identified mechanisms that help to explain how VD affects epigenetic gene regulation. Genome‐wide DNA methylation profiles in 48 healthy individuals were investigated after a 1‐month randomized isocaloric dietary intervention comparing effects of a VD versus a MR. Genome‐wide DNA methylation analysis revealed changes in differentially methylated positions following dietary intervention, with the VD group showing a higher degree of gene‐promoter silencing in cancer‐related pathways and cell growth‐associated pathways (mTOR and Hippo). Cell type deconvolution indicated an anti‐inflammatory shift in the VD group, characterized by decreased neutrophils and increased CD4^+^ T‐cell proportions, aligning with whole blood count data. Pathway analysis reflected changes in gene promoter methylation consistent with macronutrient changes according to the diets, in that VD silenced lipid metabolism and increased insulin pathway genes. Interestingly, the health‐outcome‐trained epigenetic clocks PhenoAge and GrimAge indicated a deceleration of biological aging in the VD group, while the chronologically optimized Blood&Skin clock showed an opposing acceleration. Convergent evidence from pathway analysis and health‐outcome epigenetic clocks thus suggests that a 1‐month VD drives epigenomic changes associated with reduced cancer risk and decelerated biological aging.

## Graphical abstract

This study provides comprehensive insights into the epigenetic impacts of dietary interventions, specifically contrasting the effects of a vegan diet (VD) with a meat‐rich diet (MR) on DNAm profiles and their potential health implications. Dietary habits can significantly reshape the epigenomic landscape within 1 month, with the VD exerting a more pronounced influence on potentially beneficial key biological pathways. A VD alters leukocyte composition and may promote an anti‐inflammatory state. The graphical abstract was created with BioRender.com.

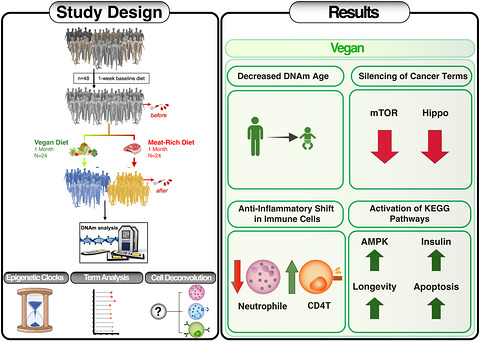

## 1 Introduction

Chronic inflammatory conditions, such as systemic autoimmune disorders, represent a significant burden on global health due to their persistent nature and the substantial healthcare resources they consume [1]. These conditions not only impair quality of life but are increasingly recognized as accelerators of systemic biological aging, contributing to the early onset of age‐related diseases and frailty, coining the term “inflammaging” [2, 3]. Potential intervention strategies aimed at slowing or reversing the molecular changes that occur with aging and to delay the onset of aging‐associated diseases cover a broad spectrum, including lifestyle changes, dietary habits, physical activity, pharmacological treatments, social interactions, and mental health support [4, 5].

Patients suffering from rheumatoid arthritis, a chronic inflammatory autoimmune disorder with over 60% of all patients aged 65 years or older, have experienced noticeable improvements on either a vegan or a quasi‐vegan diet [6, 7, 8, 9, 10]. Potential explanations include an improved ratio of n‐6 to n‐3 fatty acids, diet‐induced weight loss, and an increased intake of antioxidants [11, 12] and plant sterols inhibiting cholesterol absorption [13]. More recent studies emphasized that clinical improvements of a VD extend beyond caloric intake or weight loss and may also be attributed to regulatory effects of the diet composition and components themselves playing crucial roles in determining a diet's anti‐inflammatory potential [14, 15].

Plant‐based nutrition and VDs in particular have grown in popularity over the last two decades [16]. The VD has been associated with various health benefits, including a more favorable blood lipid profile [17], improved glycemic control [18], and systemic anti‐inflammatory effects [19, 20]. VDs may also exert beneficial effects with regard to biological aging or aging‐related chronic inflammation‐associated disorders [21, 22].

The anti‐inflammatory properties of VDs appear to be strongly influenced by their macronutrient composition and particularly their relatively low fat content [15]. While vegan diets are typically characterized by a more favorable content of phytonutrients, polyunsaturated fatty acids, and dietary fiber [23], it remains to be explored to what extent VDs modulate molecular pathways to promote homeostatic cellular states in the body and reduce pro‐inflammatory stimuli, as well as the mechanisms by which they do so. To better understand the molecular pathways linking diet to chronic inflammation and biological aging, controlled dietary studies paired with systemic profiling are required to develop therapeutic strategies aimed at mitigating their impact and improving health outcomes. In particular, epigenetic mechanisms, such as DNA methylation (DNAm), provide crucial links between nutrients, metabolites, gene regulation, and cellular states. Analyzing changes in genome‐wide DNAm profiles is therefore essential for understanding the dynamic and complex interactions between epigenetic changes and gene expression, which can offer valuable insights into disease mechanisms and potential therapeutic interventions.

A major aim of our research group is to gain a better understanding of the mechanisms mediating the effects of VDs [24, 25, 26]. In 2017, we conducted a clinical trial in 53 healthy omnivorous individuals, who were randomized to either a VD or a meat‐rich diet (MR) for 4 weeks, preceded by an initial adjustment phase consisting of a 1‐week controlled mixed diet to establish a unified baseline [24]. To control for weight loss, a common mediator in VDs, we instructed participants to maintain their total caloric intake. Our previous findings suggested that a short‐term change from a mixed diet to a vegan diet resulted in a significant reduction in neutrophilic granulocytes, monocytes, and platelets [24]. Our data also suggested an association with branched‐chain amino acids, potentially involving the mTOR (mechanistic target of rapamycin) signaling pathway.

To gain a better understanding of the mechanisms that relate to the anti‐inflammatory health benefits of VDs, we conducted genome‐wide DNAm analyses. This approach aims to uncover new insights into diet‐induced changes in molecular pathways manifested as epigenomic signatures [21]. It is widely accepted that DNAm links human phenotypes, including clinical parameters, lifestyle, and age, with epigenomic changes, which can help to identify the underlying molecular pathways [27]. In particular, epigenetic clocks trained on health‐related outcomes such as mortality risk and disease susceptibility rather than chronological age alone are especially suited to evaluate the biological impact of dietary interventions [28]. We reasoned that the aforementioned trial provides an ideal platform for DNAm analyses to explore epigenomic effects of vegan versus meat‐rich diets and to examine pathways that explain how a VD may interact with epigenetic gene regulation to promote an anti‐inflammatory systemic environment [29]. Our study was deemed well‐suited for this task as it allows for both “vegan‐versus‐meat” and for “before‐versus‐after” analyses, because it controlled for weight loss [24], and because it can be directly compared to our previous clinical reference.

## 2 Results

Plant‐based diets have been widely adopted by health‐conscious individuals as a healthier alternative to traditional diets involving animal products and may have the potential to interfere with systemic inflammatory conditions and disorders, and therefore hold promising potential as intervention strategies to increase health and life spans. Nutritional studies regarding vegan diets mostly focus on metabolic or physical readouts, while the underlying epigenomic changes remain largely understudied. To assess differences of dietary habits on the epigenomic profile of humans, we analyzed DNA methylation signatures of 48 individuals who participated in a clinical study [26]. Sample characteristics, including age, sex, and body weight, are listed in Table 1, which shows no significant differences between the groups.

**TABLE 1:** Sample characteristics.

	Vegan diet group (*n* = 24)	Meat‐rich diet group (*n* = 24)	*p*‐value
Sex			0.233^a^
Males	*n* = 7	*n* = 11	
Females	*n* = 17	*n* = 13	
Age	29 (24.50 – 42.00)	26 (22.00 – 32.50)	0.208^b^
Weight at baseline （kg）	64.75 (61.75 – 73.65	70.50 (57.80 – 80.90)	0.536^b^
Weight after dietary intervention (kg)	64.75 (61.65 – 72.80)	71.10 (58.65 – 85.25)	0.433^b^

*Note*: Sample characteristics of *n* = 48 participants.

^a^ Based on a chi‐square analyses.

^b^ Based on Mann–Whitney *U* test.

**FIGURE 1:**
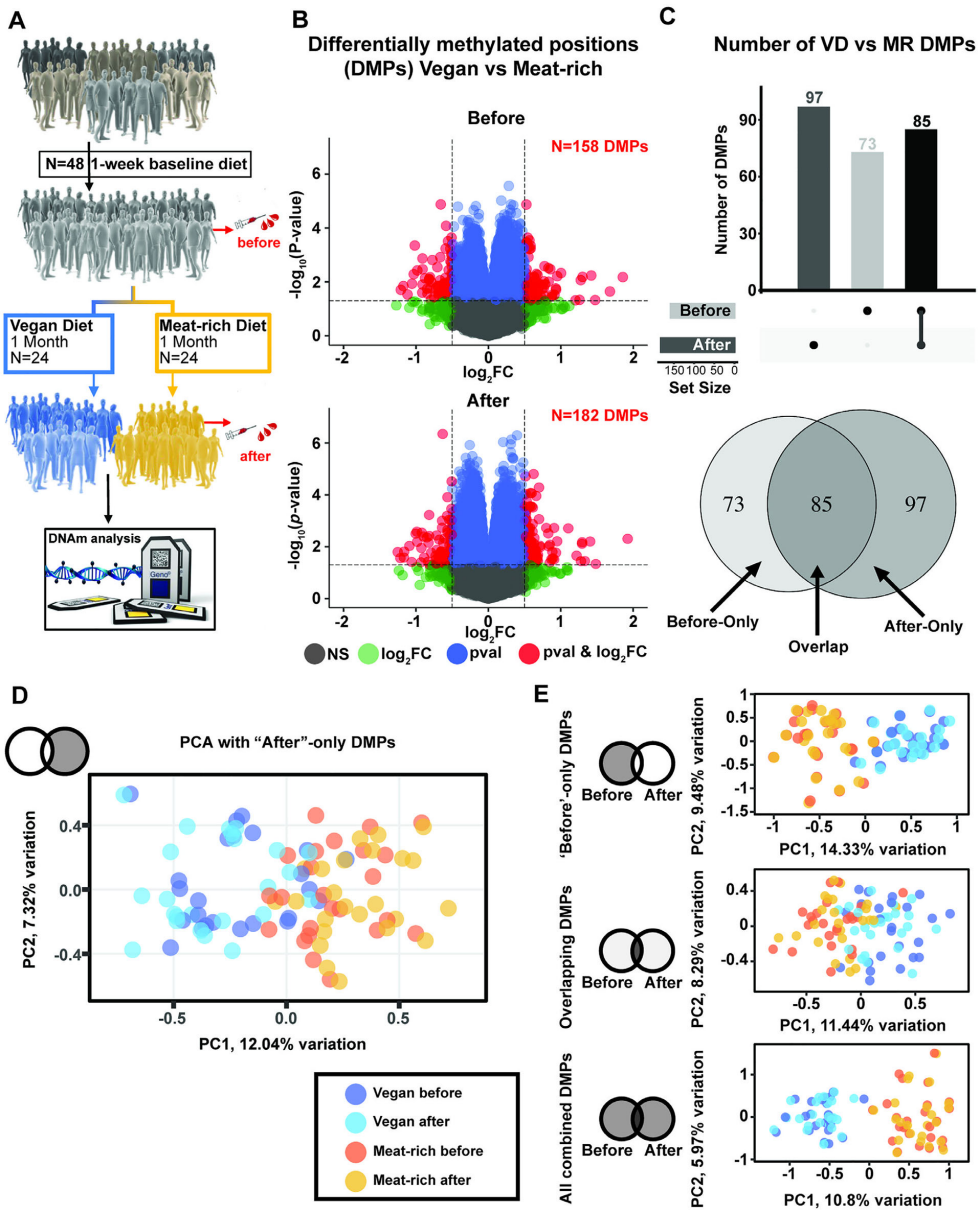
Changes in dietary habits induce epigenetic alterations of DNA CpG methylation. **(A)** Flowchart of the clinical trial setup: After an initial selection process, 48 participants were enrolled in the trial and adhered to a 1‐week dietary adjustment period to establish a unified baseline. Next, participants were randomly assigned to one of two dietary cohorts, following either a vegan diet (VD) or a meat‐rich diet (MR) for an additional month. Blood samples for DNA methylation analysis were collected at baseline and after the completion of the dietary intervention. **(B)** Volcano plot illustrating differentially methylated positions (DMPs) between the dietary cohorts both at baseline (“Before”) and at trial completion (“After”). Sites with a |log_2_FC| > 0.5 and non‐adjusted |*p*‐value| < 0.05 were deemed significantly differentially methylated and are highlighted in red. **(C)** Amount of significant DMPs (divided into intersections) found either exclusively at the “Before” or “After” time points, as well as those shared between both. **(D)** PCA of DMP beta‐values that were exclusively expressed after the trial's completion shows clear separation between dietary regimes along PC1. **(E)** PCA of DMP beta‐values that were exclusively expressed before dietary intervention (top) shows a weak separation between diets, while shared DMPs (middle) and the combination of all DMPs (bottom) show moderate separation between diets.

Participating individuals have been selected at random to follow either of two dietary regimes: a strictly VD or an MR. After an initial 1‐week period where all 48 individuals from both groups followed a standardized baseline dietary plan, blood was taken (time point: before). Subsequently, after a common baseline level was established, participants switched to a VD or MR, both of which lasted for 1 month and were concluded by drawing a second blood sample (time point: after). Next, genome‐wide EPIC array DNAm data were generated from all 96 blood samples (Figure 1A).

Sample sex predictions and SNP‐based hierarchical clustering matched the cohort data (Figure S1). Based on all quantile‐normalized CpG DNAm beta values (*n* = 812,934), hierarchical clustering showed consistent pairing of before and after samples for all but two study participants, principal component analysis (PCA) revealed no outliers or global genomic DNAm effects based on time point or diet, and no significant changes in mean global DNAm abundance were apparent between the groups (Figure S1). To probe for differences in the epigenetic DNAm profiles between both dietary groups, we calculated differentially methylated positions (DMPs) between VD and MR samples separately for before and after time points; thresholds were set to *p*‐value < 0.05 and |log_2_FC| > 0.5, but because adjusting for multiple testing by the Benjamini–Hochberg method did not yield any significant DMPs, we expanded our analysis to non‐adjusted *p*‐values that met the aforementioned thresholding criteria. This revealed 182 DMPs after the conclusion of the dietary intervention trial, while 158 DMPs were already present between participants at baseline. This marks a net increase of 24 DMPs (approximately 15%) after the dietary intervention compared to baseline levels (Figure 1B). Out of the total number of identified DMPs, 97 DMPs were uniquely differentially expressed between VD and MR after the intervention, but also more than half of the 158 DMPs observed before remained differentially methylated in the after comparison (Figure 1C). Consistently, PCA analysis based on after‐only DMPs discriminated between VD and MR after from before samples (Figure 1D,E). The paired measurements of each participant showed the greatest degree of similarities, indicating the individual methylation profile as the strongest driver of epigenetic similarity regardless of dietary change. These findings suggest that while there are measurable effects of dietary interventions on DNAm levels, inherent epigenetic variability within the cohort may occlude the diet‐induced changes in DNA methylation. The substantial inter‐individual differences complicated the ability to distinctly attribute these changes to the dietary strategies alone, making interpretation of unbiased differential data challenging and prompting more targeted analyses.

**FIGURE 2:**
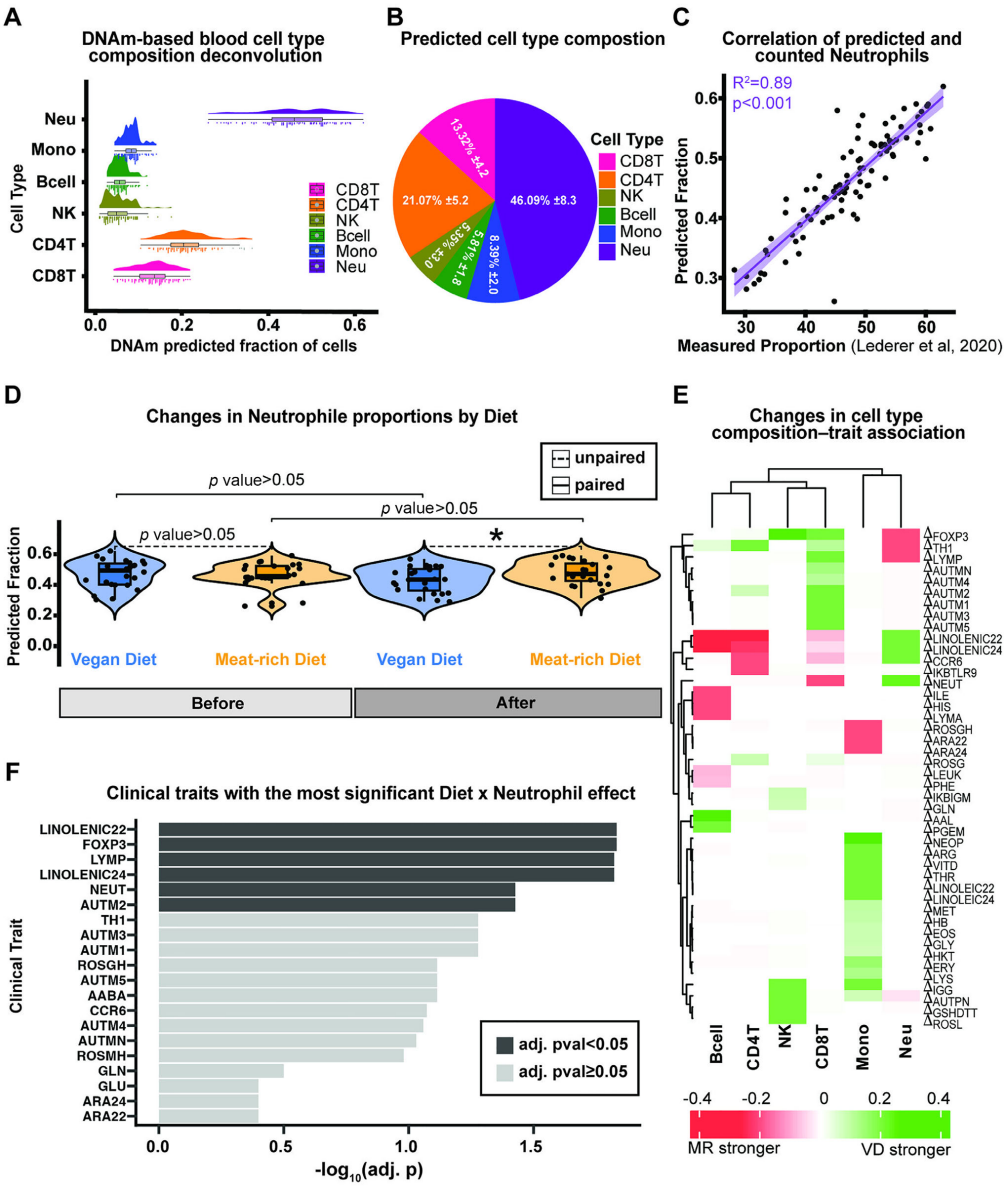
Estimation of blood leukocyte fractions through DNA methylation cell type deconvolution reveals dietary‐based changes in neutrophil counts. **(A)** Distribution of predicted leukocyte cell types inferred from CpG methylation in all tested participants (*N* = 96 samples from 48 participants, paired before‐after). **(B)** Mean predicted fractions of leukocyte cell types inferred from CpG methylation (*N* = 96 samples from 48 participants, paired before‐after). **(C)** Predicted neutrophil fraction accurately matches neutrophil counts from blood samples [24] by linear regression (*N* = 96, two paired samples from each participant). **(D)** Predicted neutrophil composition is significantly elevated in MR participants compared to VD after dietary intervention. (Welch's unpaired two‐sided *t*‐test, *p < 0.05, VD: *N* = 24, MR: *N* = 24). Inter‐group comparison between diets was conducted using a paired two‐sided *t*‐test (VD: N = 24, MR: N = 24). **(E)** Heatmap depicting if changes in leukocyte composition are differentially associated with changes in measured clinical traits during VD or MR diets to identify which immune cell types drive diet‐specific trait responses. Positive beta‐values indicate a stronger immune‐trait coupling in VD and negative with MR (VD: *N* = 24, MR: *N* = 24). **(F)** Top 20 diet‐specific interaction effects between predicted neutrophil proportion changes and clinical trait changes.

Because DNAm data from blood constitutes a heterogonous assembly of cell types, differences in the proportions of immune cells present in the blood can be indicative of physiological changes relevant to inflammation and aging. We thus sought to characterize the distribution of leukocytes from methylation signatures by performing cell type composition deconvolution (Figure 2A). Across all participants, neutrophiles were predicted to account for almost half of the leukocytes population (46.09 ± 8.3%), followed by CD4^+^ and CD8^+^ T cells (21.07 ± 5.16% and 13.32 ± 4.20%, respectively). Monocytes, B cells, and natural killer (NK) cells together accounted for less than 20% of the population (Figure 2A,B; Figure S2).

Reassuringly, comparison of the inferred proportion of neutrophiles and monocytes from DNAm with laboratory count data of the respective cell types from the blood samples reported previously [24] showed high degrees of correlation (Figure 2C, Figure S2). This strong correlation between neutrophil and monocyte counts and the DNAm‐based predictions validates the accuracy of our cell‐type deconvolution methods. This confirms the reliability of using DNAm data to estimate leukocyte composition and demonstrates the method's ability to detect diet‐induced changes in immune cell distribution. In fact, comparing predicted leukocyte composition from before and after dietary intervention revealed lower numbers of neutrophiles in the VD cohort compared to MR after intervention, but not before. Additionally, it showed a visible, though non‐significant, drop in these pro‐inflammatory leukocytes within the VD group after just 1 month (Figure 2D). The resulting change in leukocyte composition associates with changes in circulating metabolites within the blood. Notably, FOXP3 (Forkhead‐Box‐Protein P3) had a weaker association with predicted neutrophil proportion in VD (Figure 2E) and was significantly affected by changes in neutrophiles and diet (Figure 2F). FOXP3 is a well‐known master transcription factor for regulatory T cells and plays an important role in inhibiting inflammation [30, 31]. The association with linolenic acid, an anti‐inflammatory fatty acid that increased with the VD, also warrants further investigation.

Further, the level of CD4^+^ T cells was also significantly elevated in VD compared to MR (Figure S2). While previous whole blood count data did not discriminate T cell types, the drop in neutrophils and elevation of CD4^+^ T cells in the VD group compared to the MR group suggest a potential shift toward an anti‐inflammatory state, as CD4^+^ T cells are crucial in regulating immune responses and promoting immune homeostasis. These findings confirm and substantially extend previous observations of an acute diet‐induced anti‐inflammatory effect. They also provide further confidence in our genome‐wide DNAm data to probe for underlying molecular epigenetic mechanisms mediating the observed changes in blood cell composition.

**FIGURE 3:**
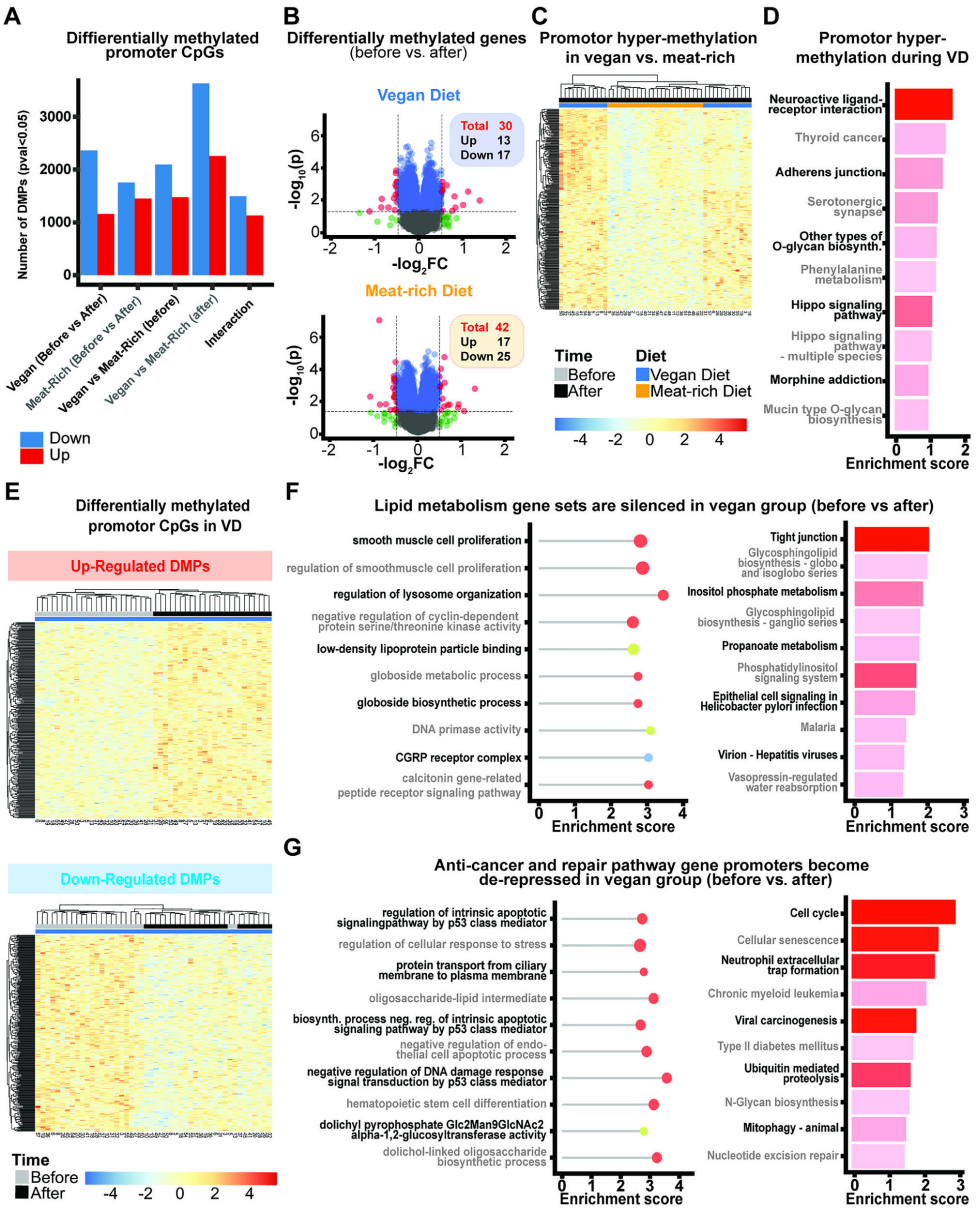
Differentially methylated promotor‐associated CpGs reveal dietary effects on metabolic and cancer‐associated pathways. **(A)** Bar plot illustrating the number of significantly up‐ and downregulated differentially methylated positions (DMPs) associated with promoters based on non‐adjusted *p*‐values (*p‐value < 0.05). **(B)** Volcano plots showcasing DMPs from before to after dietary intervention in both VD (*N* = 48 samples from 24 participants, paired before–after) and MR (*N* = 48 samples from 24 participants, paired before‐after) groups. The textbox highlights the number of CpGs meeting the significance criteria (non‐adjusted *p*‐value < 0.05 and |LFC| > 0.5). **(C)** Heatmap displaying the 2000 most upregulated β‐values of promotor‐associated CpGs after dietary intervention, revealing upregulation of promoter‐associated CpGs in VD compared to MR. **(D)** Top 10 KEGG pathways associated with upregulation in VD compared to MR following dietary intervention identified through over‐representation analysis of the 2000 most upregulated CpGs. **(E)** Heatmap depicting the 2,000 most significant beta‐values of promotor‐associated CpGs in VD, showing hyper‐methylation/gene‐silencing (top) and hypo‐methylation/gene‐activation (bottom) after 1 month of vegan diet. **(F)** Top 10 Gene Ontology (GO) terms and KEGG pathways associated with promoter‐linked CpG hyper‐methylation during 1 month of a vegan diet, determined through over‐representation analysis of the 2000 most significant *p*‐values. **(G)** Top 10 GO terms and KEGG pathways associated with promoter‐linked CpG hypo‐methylation during 1 month of vegan diet, determined through over‐representation analysis of the 2000 most significant *p*‐values.

We next focused on changes in diet‐based DNAm changes after trial completion compared to baseline. This strategy allowed for a higher degree of separation (Figure 3A,B, Figure S3), raising confidence in the approach not being skewed by individual variation. To obtain mechanistic insights into diet‐induced changes, it is important to note that the vast majority of genome‐wide DNAm sites are not linked to changes in gene expression. In fact, the sheer abundance of non‐gene‐associated CpGs in the genome can obscure significant epigenetic changes that are predictive of gene expression. We thus restricted our analysis of CpGs associated with gene promotor regions. Importantly, the number of hypo‐ and hyper‐methylated promotor‐associated DMPs and associated genes were elevated in both VD and MR groups following the change of dietary regime (Figure 3A,B, Figure S3), which is consistent with the increased number of global DMPs after the intervention (Figure 1B). Specifically, the number of hypo‐methylated promotor‐associated DMPs, indicative of diet‐induced gene activation, was higher in the MR group (Figure 3B).

Motivated by the observed changes in gene‐promotor DMP methylation and the change in leukocytes composition, we next sought to determine the biological function of the DMPs affected by diet. We first explored hyper‐methylated (silenced) genes in VD versus the MR group after the intervention (Figure 3C). Hypergeometric gene ontology (GO) over‐representation analysis identified that VD‐silenced genes were associated with cell signaling and cancer‐related molecular pathways such as “Thyroid cancer” and “Hippo signaling” (Figure 3D). To better understand the changes due to VD, we assessed enriched pathways in genes that become hyper‐methylated (silenced) or hypo‐methylated (activated) during the 1‐month VD dietary period (Figure 3E–G). Silenced genes during the VD period were primarily associated with several lipid metabolism pathways, which corresponds to the macronutrient composition of the VD (Figure 3). Further pathways related to cellular stress and homeostasis and muscle cell proliferation became epigenetically silenced during VD (Figure 3F). Activated genes during the VD period were enriched for anti‐cancer (apoptosis, senescence, stress response, cell cycle regulation) pathways, oligosaccharide metabolism pathways, and also repair and homeostasis pathways (DNA repair, ubiquitinoylation) appeared de‐repressed (Figure 3G). Overall, the observed epigenetic signatures relate to changes in diet (VD contains more carbohydrates and less fat than baseline or MR diets). Furthermore, the association of hyper‐methylated genes in the VD group with cancer‐related pathways may suggest that a VD could confer protective effects against cancer conferred by acute (1‐month) epigenetic and transcriptional changes. These observations align with evidence that plant‐based diets are linked to reduced cancer risk when compared to MRs [17, 32, 33, 34].

**FIGURE 4:**
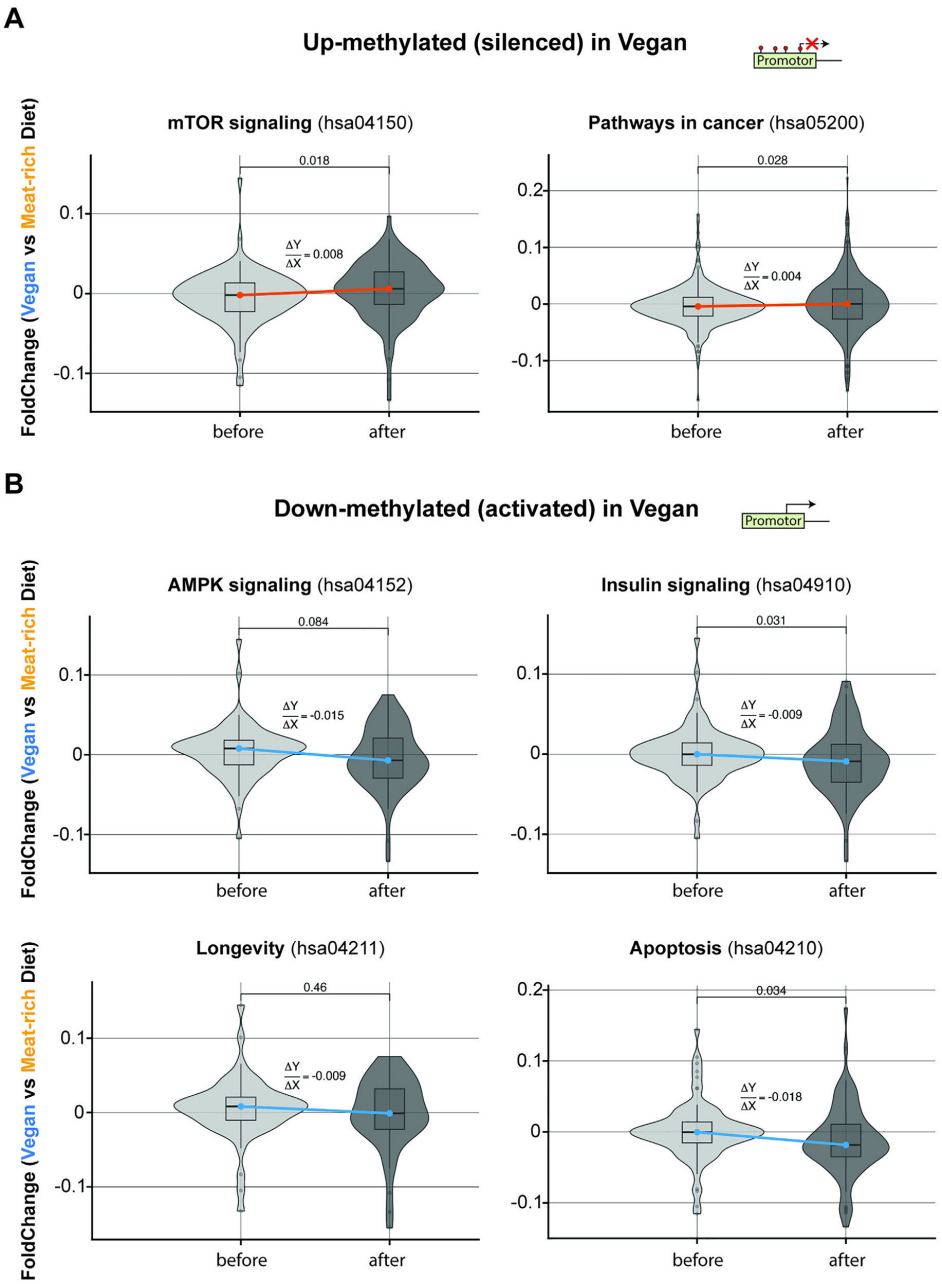
Vegan diet silences genes associated with cancer and activates metabolic and longevity pathways. **(A)** Mean promoter methylation of genes within selected KEGG pathways increases during vegan diet, associated with gene silencing of metabolic and cancer‐associated pathways. **(B)** Mean promoter methylation of genes within selected KEGG pathways decrease during vegan diet, associated with the activation of longevity and metabolism pathways.

To characterize key metabolic and cellular pathway changes on the epigenetic level, we next compared the beta‐values of CpGs belonging to selected major pathways between VD and MR that were indicated by our differential analysis. Consistently, genes belonging to “mTOR signaling” and “Pathways in Cancers” pathways were found hyper‐methylated (silenced) in VD versus MR due to dietary change and compared to baseline (Figure 4A). Conversely, stress, senescence, and survival‐promoting KEGG pathways, such as “AMPK signaling,” “Insulin signaling,” “Longevity,” and “Apoptosis,” exhibit a loss of methylation signatures, indicative of their activation in the VD hypo‐methylated cohort post‐intervention (Figure 4B).

Because changes in diet previously evoked changes in measured metabolites within the blood of human study participants (Lederer et al., Figure S3D), we next sought to determine the effect methylation has on these diet‐specific differences. We analyzed the diet‐specific modulation of DNA methylation and measured plasma constituents. The diet‐associated differences largely aligned with previously measured clinical data, for example, higher serum concentrations of various amino acids including valine and lysine in MR participants, higher vitamin B12 status‐related markers in MR participants as well as higher glycine levels in VD participants. In the methylation–trait relationship analysis, we examined how differences in DNA methylation patterns were linked to differences in clinical traits (Figure S3E). Again, a potential role for FOXP3 in vegans emerged, whereas the aforementioned amino acids apparently played no role in said context.

**FIGURE 5:**
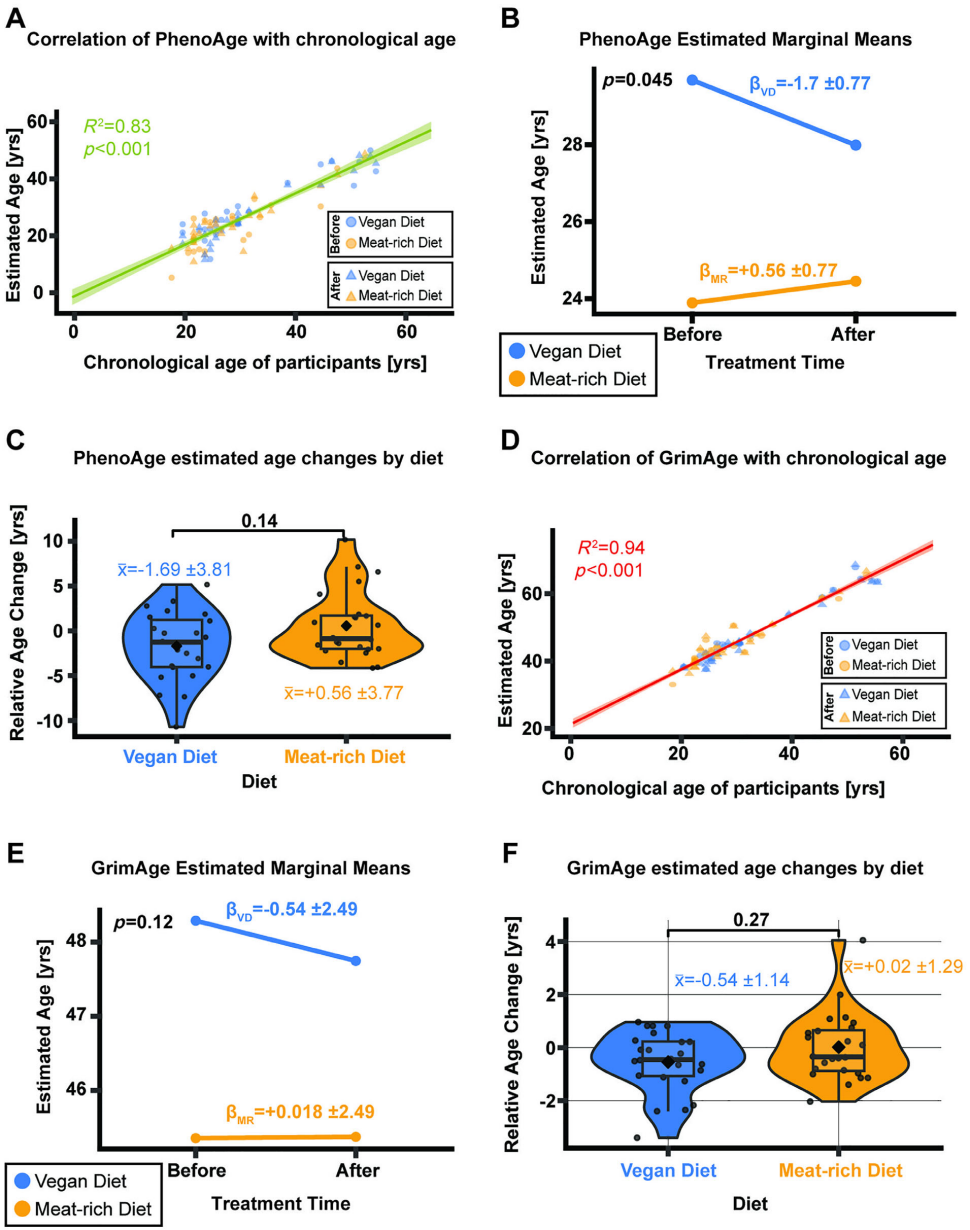
Methylation clock analysis reveals changes in epigenetic aging based on diet. **(A)** The Levine PhenoAge clock shows a high correlation between estimated methylation‐based age and actual chronological age of all samples (*N* = 96). **(B)** Estimated marginal means of Levine PhenoAge DNA methylation age prediction from a linear mixed effect model using Diet × Time as fixed effects, and participant as a random effect: (Diet × Time, *p* = 0.045) (VD: *β* = −1.69, 95% CI [−3.25, −0.13], *N* = 24; MR: *β* = +0.56, 95% CI [−1.0, +2,12], *N* = 24). **(C)** Baseline‐normalized DNAm‐scores predicted by Levine's clock (PhenoAge) show a slight but not significant decrease in age of the vegan cohort compared to MR (*N* = 24 per group). Points represent estimated relative age per donor; diamond represents the mean value. **(D)** The GrimAge clock shows a high correlation between estimated methylation‐based age and actual chronological age of all samples (*N* = 96). **(E)** Estimated marginal means of GrimAge DNA methylation age prediction from a linear mixed effect model, using Diet × Time as fixed effects, and participant as a random effect (Diet × Time, *p* = 0.12), (VD: β = −0.54, 95% CI [−1.04, −0.04], *N* = 24; MR: β = +0.02, 95% CI [−0.48, +0.52], *N* = 24). **(F)** Baseline‐normalized DNAm‐scores predicted by GrimAge shows no significant decrease in age of the vegan cohort compared to MR (*N* = 24 per group). Points represent estimated relative age per donor; diamond represents the mean value.

Because systemic inflammatory signatures and disorders are highly age dependent, and because many of the pathways that change due to diet are associated with inflammation, aging, and longevity, we next assessed differences in biological age between MR and VD strategies. Epigenetic clocks (DNAm clocks) are algorithms that estimate a “biological age” from CpG methylation signatures and can further serve as a measurement of overall health, when compared to chronological age (age acceleration/deceleration) [35]. We calculated 10 different DNAm clocks for our samples, and focused our primary interpretation on clocks specifically trained to predict health‐related outcomes rather than chronological age (Figure S4) [36, 37, 38]. The PhenoAge clock, specifically designed to estimate biological age‐related health outcomes such as age‐related disease susceptibility [36, 38], showed robust accuracy in predicting chronological age in our cohort (*R* ^2^ = 0.83; Figure 5A), confirming its reliability in our sample without being exclusively optimized for pure chronological age tracking. Critically, PhenoAge revealed a significant diet‐dependent divergence in biological aging trajectories: the Diet × Time interaction revealed a significant divergence between diets (*p* = 0.045), with estimated marginal means showing a significant deceleration in VD (slope = −1.7 years, 95% CI [−3.25, −0.13], *N* = 24) and an opposing increasing trend in MR (slope = +0.56 years 95% CI [−1, 2.12], *N* = 24; Figure 5B). Direct between‐group comparison of PhenoAge age acceleration estimates after the intervention did not reach statistical significance (VD: −1.69 years vs. MR: +0.56 years; *p* = 0.14) at this cohort size (Figure 5C).

This finding was directionally supported by GrimAge, an independent clock developed to predict mortality risk and overall healthiness, which also showed high accuracy in predicting chronological age (*R* ^2^ = 0.94) [39]. While the Diet × Time interaction did not reach statistical significance (*p* = 0.12), estimated marginal means also showed a significant decrease of estimated age during treatment in those assigned to VD (slope = −0.53 years, 95% CI [−1.04, −0.04], *N* = 24); it was not significantly changed in those assigned to MR (slope = +0.018 years, 95% CI [−0.48, 0.52], *N* = 24; Figure 5E). Direct between‐group comparison of GrimAge age acceleration estimates after the intervention showed the same directionality as the PhenoAge clock, but was not significant (VD: −0.54 years vs. MR: +0.02 years; *p* = 0.27) at this cohort size (Figure 5F).

In contrast, the Blood&Skin clock, which achieves the highest accuracy in predicting chronological age among all tested clocks (*R* ^2^ = 0.95; Figure S4), showed an opposing pattern. The Diet × Time interaction revealed a significant divergence between diets (*p* = 0.008), with estimated marginal means showing an acceleration of predicted age in those assigned to VD (slope = +0.61 years, 95% CI [−0.02, 1.23], *N* = 24) and a deceleration in those assigned to MR (slope = −0.61 years, 95% CI [−1,23, 0.01], *N* = 24; Figure S4). Direct between‐group comparison of baseline‐corrected Blood&Skin estimates after the intervention confirmed a significantly higher predicted age acceleration in VD compared to MR (+0.61 years vs. −0.61 years; *p* = 0.0059; Figure S4).

Taken together, the convergent signals from the two health‐outcome‐trained clocks, PhenoAge and GrimAge, indicate that a 1‐month VD is associated with a reduction in overall health‐related epigenetic age, which is consistent with other anti‐aging benefits of plant‐based diets [22].

## 3 Discussion

This study provides comprehensive insights into the epigenetic impacts of dietary interventions, specifically contrasting the effects of a VD with an MR diet on DNAm profiles and their potential health implications. Our findings reveal that dietary habits can significantly reshape the epigenomic landscape within 1 month, with the VD exerting a more pronounced influence on potentially beneficial key biological pathways.

The increase in DMPs following dietary intervention highlights the potential of diet to modulate epigenetic marks. Notably, the VD cohort exhibited a higher degree of gene‐promoter silencing in pathways associated with cell signaling and cancer, such as the Hippo signaling pathway. This observation aligns with the existing literature, suggesting that plant‐based diets may confer protective effects against cancer by modulating epigenetic regulation of oncogenic pathways [40, 41]. These findings are also consistent with previous studies indicating that plant‐based nutrition is associated with reduced cancer risk and improved metabolic profiles [17, 32, 33, 34, 42]. The Hippo signaling pathway, for example, plays an important role in the regulation of tissue homeostasis and cell growth/proliferation control [43]. As reviewed by Kong et al., this signaling pathway is involved in the regulation of immune cells, such as macrophages, T cells, and dendritic cells, and further interacts with immune‐related pathways such as NF‐kB—all of which play an important role in autoimmune disorders [44]. Since the Hippo pathway possesses the capacity to lead to tumorigenesis [45], it appears of utmost importance that a short‐term vegan intervention may epigenetically influence it. Notably, we are not the first group to highlight this association. The fact that a VD may alter the Hippo pathways has previously been suggested by a study by Filippov et al., who used data from the Adventist Health Study II with a high number of vegetarian and vegan participants [29]. Our data reinforce these findings and thus emphasize a potential role for VDs in cancer and autoimmune disorders.

Our study also highlights the complex interplay between diet, epigenetic regulation, and the dual roles of these pathways in health and disease. It suggests that while plant‐based diets may activate protective anti‐cancer pathways, the short‐term epigenomic response to VD produces divergent signals depending on which aspect of aging is measured: health‐outcome‐trained clocks indicate a deceleration in predicted biological age, while the chronologically optimized Blood&Skin clock suggests the opposite. This complexity necessitates a balanced approach to dietary recommendations and a focus on whole‐food plant components. The differential impact on leukocyte composition further supports the notion that a VD may promote an anti‐inflammatory state. Again, these findings align well with the previous literature on whole blood count data in vegans [46, 47, 48].

The reduction in neutrophils and increase in CD4^+^ T cells observed in the VD cohort in the present study suggest a shift toward immune homeostasis, potentially mitigating systemic inflammation associated with aging. This observed increase in CD4^+^ T cells aligns well with a recently published study reporting an increase in the levels of activated CD4^+^ T cells upon a VD intervention [49]. One potential explanation might be the abrupt decrease in the consumption of animal products [49]. Processed foods (e.g., granola bars, high fructose corn syrup, and sweets with a high fat content), however, could also play a pivotal role in this context [49]. These foods were permitted in the vegan group in our study to maintain a constant energy intake and to prevent weight loss. The fact that a lacto‐ovo‐vegetarian dietary intervention, as reported by Richter et al., did not affect the number of CD4^+^ cells supports the potential role of animal products in this context [50]. The predicted increase in CD4^+^ T cells in our study aligns with the concept of “inflammaging,” where chronic inflammation accelerates biological aging and contributes to age‐related diseases [51]. Our findings suggest that dietary interventions, particularly plant‐based diets, could play a crucial role in modulating immune responses and dampening systemic inflammation, thereby potentially delaying the onset of aging‐related diseases.

The divergent signals between epigenetic clocks observed in our study reflect a fundamental distinction in what these clocks measure. The Blood&Skin clock, optimized to track chronological age [28], showed accelerated predicted age in the VD group—consistent with its sensitivity to diet‐induced epigenetic dynamics in CpG sites that covary with chronological aging rather than health. In contrast, PhenoAge and GrimAge, both trained on health‐related outcomes, consistently indicated a trend toward deceleration of biological aging in the VD group. The activation of protective pathways in the VD group, such as AMPK signaling, apoptosis, and senescence, may contribute to these divergent signals: while such pathways can alter CpG methylation patterns tracked by chronological clocks, they represent established anti‐cancer and longevity‐promoting mechanisms that are appropriately captured by health‐outcome clocks as biological aging benefits. Further, our findings are consistent with those of Dwaraka et al., who reported decreased epigenetic age acceleration in a vegan group using PhenoAge, GrimAge, and DunedinPACE, all health‐outcome‐trained clocks, alongside increased telomere length [21]. Critically, Dwaraka et al. did not report results for the Horvath Blood&Skin, and the apparent discrepancy between our Blood&Skin result and our PhenoAge/GrimAge and Dwaraka's findings likely reflects the use of chronologically versus health‐optimized clocks. Where clock designs are comparable, our results converge with theirs. A key difference between both studies is that Dwaraka et al. emphasized whole plant foods and allowed for weight loss in their study. Our study did not specifically investigate telomere length, but it is well known that comprehensive lifestyle changes centered around plant‐based nutrition may have a significant effect in this context [52]. Our findings highlight the potential of the health‐centered epigenetic clocks as valuable tools for assessing the impact of dietary interventions on biological aging and health outcomes, and underscore the need for further research with longer dietary intervention periods and larger participant cohorts to fully understand the implications of these epigenetic changes.

Overall, our controlled short‐term study highlights the nuanced effects of dietary interventions on the epigenome and their broader implications for health and longevity. While the chronologically optimized Blood&Skin clock suggests short‐term acceleration in predicted chronological age, health‐outcome clocks indicate the. Future research should aim to disentangle these complex interactions and explore strategies to optimize dietary interventions for balanced health outcomes. It remains to be explored whether this could be the effect of an unsupplemented VD devoid of vitamin B12 and other nutrients that are mainly derived from animal foods [21]. Epigenetic analyses based on a follow‐up study of our trial with a longer duration will soon be performed [53]. Future studies should investigate the long‐term effects of dietary patterns on epigenetic regulation and identify specific dietary components that may mitigate the accelerated chronological aging as captured by the Blood&Skin clock, while enhancing anti‐cancer benefits. By advancing our understanding of the molecular mechanisms linking diet to health, we can develop targeted dietary strategies that delay onset of aging‐related disorders, and thus promote health and longevity.

Finally, it is noteworthy that genes belonging to the “mTOR signaling” (KEGG) pathways were found hyper‐methylated (silenced) in VD versus MR. Melnik extensively reviewed the consequences of a Western‐diet driven mTORC1 hyperactivation, including increased cell growth, proliferation, and protein synthesis [54, 55]. Given that mTORC1 is “the” master‐switch for eukaryotic cell growth, our findings are of significant importance and warrant further investigation in future studies [55].

The present study has strengths and limitations that warrant consideration. As for the strengths, we present data from an innovative prospective, randomized clinical dietary intervention with a control group. The pre‐specified inclusion and exclusion criteria may have played an important role in recruiting healthy individuals, so that the results emphasize that dietary behaviors play an important role in disease prevention. We carefully controlled for weight loss and meticulously instructed participants to maintain a constant daily energy intake. This clinical adjustment for an important confounder may have far‐reaching consequences. While our data suggest that diet‐related epigenetic modulations may occur independent of weight loss, one must also acknowledge that adjusting for weight loss may translate into mediator adjustment from a statistical point of view. The VD in our study was designed with an isocaloric character in mind, which implied that some participants artificially consumed higher amounts of nuts, oils or granola bars to achieve the daily energy intake target of 1800–2000 kcal/day. Furthermore, the VD in this study mainly excluded all animal products while no special emphasis was paid to maximizing the intake of unprocessed, whole plant foods. Finally, we acknowledge the small sample size and the rather short duration of the dietary intervention, which yielded no significant *p*‐values after multiple correction.

Those factors, together with the high number of tested CpG sites, could explain the absence of significant epigenetic changes, since we predict the evoked changes as small and a larger cohort would be required to detect small between group differences.

Despite these limitations, this study provides novel insights into the molecular mechanisms underlying the health effects of vegan diets and motivates further research to optimize dietary interventions for balanced health outcomes and longevity. The higher degree of gene‐promoter silencing in cancer‐related pathways and cell growth‐associated pathways (mTOR and Hippo) in the VD group following the dietary intervention is particularly noticeable and may pave the way for targeted dietary interventions.

## 4 Materials and Methods

### 4.1 Study Population and Design

This is a secondary analysis of a monocentric, randomized‐controlled clinical pilot trial with a parallel group design, which aimed to evaluate the effects of a short‐term isocaloric vegan dietary intervention on the immune system and metabolism of healthy free‐living individuals. Meat‐rich participants (consuming > 150 g meat/day) served as a control group. The methods and study characteristics have been described elsewhere in detail [24, 25, 26]. In brief, we enrolled healthy omnivorous individuals aged 18–60 years, without clinically relevant allergies or chronic health conditions. Eating disorders, smoking, participation in another clinical trial, and blood donations within 4 weeks prior to the study enrollment yielded reasons for ineligibility. Additional exclusion criteria included abuse of drugs or alcohol as well as a regular intake of medication. Individuals consuming any form of plant‐based diet (e. g. vegetarian or vegan) prior to the study were not considered eligible. The trial was approved by the ethics committee of the University Medical Center of Freiburg, Germany (EK Freiburg 38/17_170329) before onset and was registered at the German Clinical Trial Register (DRKS00011963). The entire study was performed in accordance with the principles of the Declaration of Helsinki and the guidelines of ICH (International Conference on Harmonization) for good clinical practice. All participants provided written and oral informed consent before participation.

### 4.2 Blood DNA Isolation and Genome‐Wide DNA Methylation Analysis

Fasting blood samples were collected at our department at the Medical Center‐ University of Freiburg as described previously in detail [26]. Notably, while our original study comprised *n* = 53 individuals, isolated DNA was only available for *n* = 48 individuals and was drawn at two time points, before (t0/before) and 1 month after (t1/after) switching to a VD/MR. Genomic DNA was isolated from whole blood samples, obtained under sterile conditions, by precipitation [56], and genome‐wide DNAm analysis was carried out using the Illumina Infinium MethylationEPIC BeadChip array at the Human Genomics Facility of the Genetic Laboratory of the Department of Internal Medicine at Erasmus MC University Medical Center Rotterdam. The DNA was isolated in 2019, and the DNAm analysis was performed in 2021 and 2022.

### 4.3 Processing of Methylation Data

Raw IDAT files from the Illumina EPIC array analysis were processed using the Minfi package (version 1.44.0) in R (version 4.2.3) to obtain beta‐ and M‐values for downstream analysis. Additional CpG annotations were sourced from the “IlluminaHumanMethylationEPICanno.ilm10b4.hg19” package, utilizing the annotation file MethylationEPIC_v‐1‐0_B4.csv from official Illumina sources. Sample quality was assessed based on detection *p*‐values < 0.05, but no samples needed to be excluded from the analysis. Poorly performing probes were filtered using a more stringent detection *p*‐value threshold of < 0.01, resulting in the removal of 5445 sites. To eliminate sex‐specific effects, we excluded 19,071 sites located on sex chromosomes. Additionally, 28,409 loci associated with common SNPs were removed, bringing the final number of sites used in the downstream analysis to 812,934. Methylation data were normalized using stratified quantile normalization within the Minfi package.

### 4.4 Differential Methylation Analysis

DMPs were identified using the limma package (version 3.54.0) in R. Individual contrasts were defined for each diet across both time points to detect before–after treatment effects in the corresponding dietary intervention. Additionally, DMPs between both dietary regimes at either timepoint were assessed by setting the corresponding contrasts. A normalized interaction effect between the diets posttreatment was calculated by subtracting the baseline effect from the post‐treatment effect before comparing the two diets directly. The original model did not explicitly control for paired data; the later adjusted models included blocking by subjects. Neither model yielded significant DMPs after Benjamini–Hochberg adjustment of *p*‐values for multiple testing—instead, raw *p*‐values and log_2_FC were used for most downstream analysis.

Promoter‐associated CpGs were identified using the “IlluminaHumanMethylationEPICanno.ilm10b4.hg19” manifest data by filtering for CpGs annotated with either of the features: “Promoter_Associated_Cell_type_specific” or “Promoter_Associated.” Average promoter methylation for each gene was calculated by taking the mean of the beta‐values associated with these features.

### 4.5 Analysis of Biological Significance

Biological functions for DMPs were inferred as GO terms or KEGG (Kyoto Encyclopedia of Genes and Genomes) pathways through mapping CpGs to the corresponding gene and using hypergeometric overrepresentation analysis (ORA) while correcting for the number of CpGs per gene by using the “MissMethyl” package's (version 0.99.0) gometh function. The 2000 CpGs with the smallest raw *p*‐values were used as input. Promotor‐associated terms were restricted to CpGs associated with the genomic features: “TSS1500,” “TSS200,” “1stExon,” and “5'UTR.” For gene set enrichment analysis (GSEA), the methylRRA function was used, providing all CpGs ranked by raw *p* value and signed according to log_2_FC as input.

### 4.6 Cell Type Deconvolution

Whole blood cell type composition was estimated from methylation data using the “minfi” package with Illumina EPIC data on immunomagnetic sorted peripheral adult blood cells from “FlowSorted.Blood.EPIC” [57]. Data were processed using the noob pre‐processing, and IlluminaHumanMethylationEPIC was used as a reference platform [58].

### 4.7 DNA Methylation Age Estimation

DNA methylation age was estimated from the previously calculated beta‐values using the “methylclock” package (version 1.4.0) [59]. Estimates were generated for all included algorithms: “Horvath,” “Hannum,” “Levine,” “BNN,” “skinHorvath,” “PedBE,” “Wu,” “TL,” “BLUP,” and “EN.” The Levine/PhenoAge Clock, GrimAge Clock, and skinHorvath/Blood&Skin Clock estimates were further analyzed due to their relevance to health‐related aging outcomes and chronological age prediction, respectively. Relative age change was defined as the difference in estimated DNA methylation age observed during the dietary intervention. It was calculated by subtracting the estimated DNAm age at baseline from the estimated DNAm age at the end of the trial. GrimAge was calculated in R using the “dnaMethyAge” (version 0.2.0) package [60]. A total of 4 of 514 (0.78%) CpGs were missing and not included in age estimation for Levine PhenoAge, 6 of 392 (1.53%) CpGs were missing and not included for Blood&Skin (skinHorvath), and 1505 of 78,465 (1.92%) were missing and not included for GrimAge.

### 4.8 DNA Methylation and Clinical Trait Association

To assess whether changes in global methylation explain variation in clinical trait changes beyond diet alone, we computed within‐subject differences for each clinical trait and M‐values. We collapsed the change in methylation and summarized it by conducting PCA. For each clinical trait, a base linear model was fit between delta trait and dietary group as well as sex and age as covariates. A second model was fit by expanding the base model with the first five methylation PCs. Both models were compared using a nested ANOVA *F*‐test followed by Benjamini–Hochberg correction for multiple testing.

### 4.9 Statistical Analysis

All statistical analyses were performed using R (version 4.2.3). Differences in mean values between time points were assessed using paired two‐sided Welch's *t*‐tests; for comparisons across diets, unpaired two‐sided Welch's *t*‐tests were used. To assess diet‐dependent changes in epigenetic clock estimates over time, linear mixed effects models were fitted for Diet, Time, and their interaction (Diet × Time) as fixed effects and participant as a random effect. Estimated marginal means were derived from these models to summarize the Diet × Time interaction effects while accounting for subject variability, enabling the quantification of the trajectory of aging within each dietary group. Models were fitted using the “lme4” (version 1.1‐37) package in R, while estimated marginal means were calculated using the “emmeans” (version 1.11.2) package.

## Author Contributions

L. Karbacher conducted research, coordinated the epigenetic analysis process, analyzed and helped interpret the results, synthesized the results, wrote the paper, and was responsible for visualization. J. Mertens conducted research, coordinated the epigenetic analysis process, analyzed and interpreted the data, wrote the paper, and had primary responsibility for final content. S. Kowarschik conducted research, provided essential materials, and coordinated the epigenetic analysis process. A. K. Lederer designed the original study/research (project conception, study oversight), conducted research, and provided essential materials. M. Ku conducted research, provided essential materials, and coordinated the epigenetic analysis process. R. Huber designed the original study/research (project conception, study oversight), conducted research, and provided essential materials. MA. Storz designed the research (project conception), conducted research, coordinated the epigenetic analysis process, wrote the paper, and had primary responsibility for final content. All authors have read and approved the final manuscript.

## Funding

The Ministry of Science, Research and Arts of the state Baden‐Württemberg, Germany, financed the position of Ann‐Kathrin Lederer within the Academic Center for Complementary and Integrative Medicine (AZKIM); otherwise, the study was financed by institutional resources. The work was further supported by the National Institutes of Health (NIH) awards R01‐AG085634, RF1‐AG056306, RF1‐AG095193, P30‐AG062429, and U19‐AG023122, the California Institute for Regenerative Medicine (CIRM) award DISC0‐17507, the BightFocus Foundation award A2024036S, and the American Federation for Aging Research (AFAR) award 30376715.

## Ethics Statement

The study was registered at the German Clinical Trial register (DRKS00011963) and approved by the ethical committee of the University Medical Center of Freiburg, Germany (EK Freiburg number: 38/17_170329) before onset. All participants provided written informed consent before participation.

## Conflicts of Interest

The authors declare no conflicts of interest.

## Supporting information




**Supplementary Figure 1**: (A) Sex prediction from methylation data accurately predicts participants reported sex (N = 96). (B) SNP‐based hierarchical clustering. (C) Quantile normalization robustly removes variation from raw data. (D) Hierarchical clustering of all participants shows clustering by participant (N = 96). (E) Global PCA analysis of all beta‐values neither shows significant separation when resolved by time (top) nor by diet (bottom) (N = 96). (F) Mean promotor methylation of all promotor‐associated sites at baseline and after treatment in VD (left, N = 48 samples, 24 paired measurements before‐after) and MR (right, N = 48 samples, 24 paired measurements before‐after).
**Supplementary Figure 2**: (A) Predicted fractions of leukocyte cell types inferred from CpG methylation in all tested participants (N = 96). (B) Predicted monocyte fraction accurately matches monocyte counts from blood samples ([24]) by linear regression (R^2^ = 0.89, pval<0.001, N = 96). (C‐D) Predicted changes in blood cell‐type composition between MR and VD participants before and after dietary intervention as grouped comparison (C) and shown for each individual (D). CD4^+^T‐cell proportions decrease in MR after intervention, while Neutrophile proportions are elevated. (Welch's two‐sided t‐test, *p<0.05, N = 24). Differences in Neutrophile composition appeared to mainly be driven by a subset of participants, indicating the presence of responders and non‐responders to the intervention.
**Supplementary Figure 3**: (A) Bar plot illustrating the magnitude of all up‐ and down‐regulated differentially methylated positions (DMPs), based on non‐adjusted p‐values (non‐adjusted pvalue<0.05, |log_2_FC| >0). (B) Intersections of significant DMPs (non‐adjusted pvalue<0.05 and |log_2_FC|>0.5) found exclusively in either the vegan or meat‐rich cohorts after the respective dietary intervention, and those shared between them. (C PCA of all DMPs found between VD and MR shows separation of samples between ‘before’ from ‘after’. (D) Volcano plot showing diet‐associated effects on changes in clinically measured traits post dietary Intervention.
**(E)** Volcano plot showing diet‐dependent differences in the association of global methylation variation and changes in clinical traits, adjusted for sex and age. Values stronger for either diet, indicate a stronger methylation‐trait relationship within that group.
**Supplementary Figure 4**: (A) Comparison of ten different DNAm algorithms from participants blood samples show various degrees of prediction accuracy in reference to the participants’ chronological age. Horvath's Skin&Blood clock and Levine's PhenoAge emerge as the most reliable models for age prediction from human blood samples (N = 96). (B) Baseline‐normalization shows a significant decrease of estimated DNAm age with Horvarth's Skin&Blood Clock (‘skinHorvarth’) in MR participants after dietary intervention, but not in other algorithms (VD: N = 24, MR: N = 24). (C) The Horvath Skin&Blood clock shows a high correlation (R^2^ = 0.95, pvalue<0.001) between estimated methylation‐based age and actual chronological age of all participants (N = 96). (D) Estimated marginal means of the Blood&Skin DNA methylation age prediction from a linear mixed effect model, using Diet x Time as fixed effects, and participant as a random effect (Diet x Time p = 0.12), (VD: β = +0.61, 95% CI [‐0.02, +1.23], N = 24; MR: β = ‐0.61, 95% CI [‐1.23, +0.01], N = 24). (E) Baseline‐normalized DNAm‐scores predicted by the Blood&Skin clock shows a significant decrease in age of the MR cohort compared to VD (VD: N = 24, MR: N = 24). Points represent estimated relative age per donor; Diamond represents the mean value. (F) Line plots showing the estimated age of the VD cohort (top) and MR cohort (bottom) from before to after treatment for each participant as predicted by the PhenoAge, GrimAge, and Blood&Skin estimators. (G) Summary of F showing the amount of participants that either experience increase or decrease in estimated age during dietary intervention.

## Data Availability

The genome‐wide DNA methylation data generated in this study have been submitted to the ArrayExpress repository (EMBL‐EBI) The accession number is E‐MTAB‐17025.
