# Supplementary material for: A Vegan Diet Epigenetically Modulates Inflammatory Pathways and Biological Aging: Genome‐Wide DNA Methylation Analysis of a One‐Month Isocaloric Vegan Versus Meat‐Rich Dietary Intervention
Source: MedComm (2020). 2026 Aug 3;7(8):e70899. doi: 10.1002/mco2.70899 (PMC13430503; doi:10.1002/mco2.70899)
Supplement: Supplementary file 1 — Supplementary Figure 1: (A) Sex prediction from methylation data accurately predicts participants reported sex (N = 96). (B) SNP‐based hierarchical clustering. (C) Quantile normalization robustly removes variation from raw data. (D) Hierarchical clustering of all participants shows clustering by participant (N = 96). (E) Global PCA analysis of all beta‐values neither shows significant separation when resolved by time (top) nor by diet (bottom) (N = 96). (F) Mean promotor methylation of all promotor‐associated sites at baseline and after treatment in VD (left, N = 48 samples, 24 paired measurements before‐after) and MR (right, N = 48 samples, 24 paired measurements before‐after). Supplementary Figure 2: (A) Predicted fractions of leukocyte cell types inferred from CpG methylation in all tested participants (N = 96). (B) Predicted monocyte fraction accurately matches monocyte counts from blood samples ([24]) by linear regression (R2 = 0.89, pval<0.001, N = 96). (C‐D) Predicted changes in blood cell‐type composition between MR and VD participants before and after dietary intervention as grouped comparison (C) and shown for each individual (D). CD4+T‐cell proportions decrease in MR after intervention, while Neutrophile proportions are elevated. (Welch's two‐sided t‐test, *p<0.05, N = 24). Differences in Neutrophile composition appeared to mainly be driven by a subset of participants, indicating the presence of responders and non‐responders to the intervention. Supplementary Figure 3: (A) Bar plot illustrating the magnitude of all up‐ and down‐regulated differentially methylated positions (DMPs), based on non‐adjusted p‐values (non‐adjusted pvalue<0.05, |log2FC| >0). (B) Intersections of significant DMPs (non‐adjusted pvalue<0.05 and |log2FC|>0.5) found exclusively in either the vegan or meat‐rich cohorts after the respective dietary intervention, and those shared between them. (C PCA of all DMPs found between VD and MR shows separation of samples between ‘before’ from ‘ [file MCO2-7-e70899-s001.docx]

**A vegan diet epigenetically modulates inflammatory pathways and biological aging: Genome-wide DNA methylation analysis of a one-month isocaloric vegan versus meat-rich dietary intervention.**

Karbacher L ^1,2^, Mertens J ^1,2,*^, Kowarschik S ^3^, Lederer AK ^3,4^, Ku M ^5^, Huber R ^3^, Storz MA ^3,*^

^1^ Department of Neurosciences, University of California San Diego, La Jolla, CA, USA

^2^ Laboratory of Genetics, The Salk Institute for Biological Studies, La Jolla, CA, USA

^3^ Department of Internal Medicine II, Center for Complementary Medicine, Medical Center - University of Freiburg, Faculty of Medicine, University of Freiburg, Germany

^4^ Department of General, Visceral and Transplantation Surgery, University Medical Center Mainz, Mainz, Germany

^5^ Department of Pediatrics and Adolescent Medicine, Division of Pediatric Hematology and Oncology, Faculty of Medicine, Medical Center - University of Freiburg, Freiburg, Germany

Corresponding authors:

Maximilian Andreas Storz, Department of Internal Medicine II, Center for Complementary Medicine, Medical Center - University of Freiburg, Faculty of Medicine, University of Freiburg, Germany. E-mail: maximilian.storz@uniklinik-freiburg.de, Phone: +49 15754543852; ORCID-ID: 0000-0003-3277-0301

Jerome Mertens, Department of Neurosciences, UC San Diego, Sanford Consortium for Regenerative Medicine, 2880 Torrey Pines Scenic Drive, La Jolla, CA 92037, jmertens@health.ucsd.edu; ORCID-ID: 0000-0002-4291-4121

Running head: Epigenetic impact of vegan vs. meat-rich diet

**Supplementary Figure 1:**


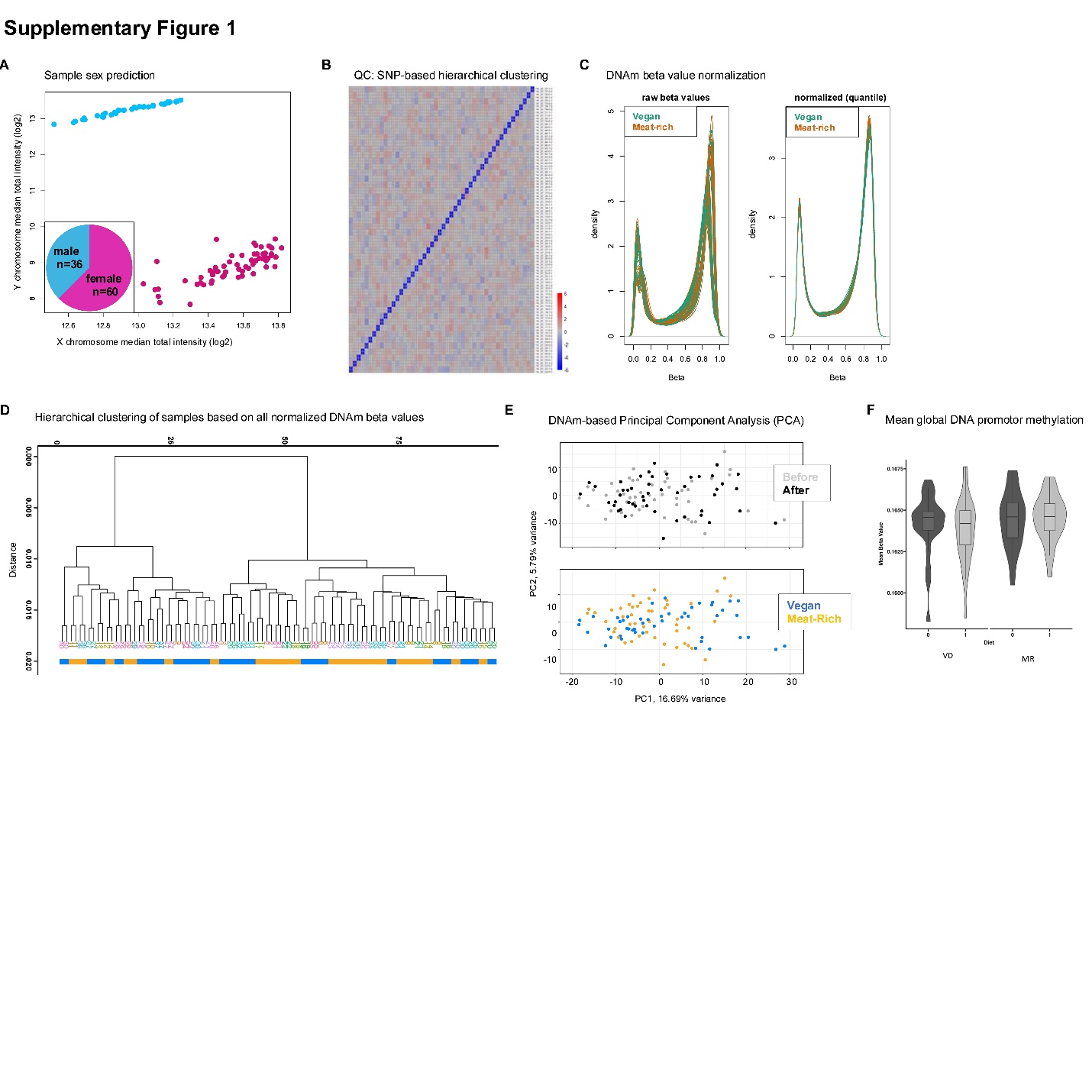


**Supplementary Figure 1 legend:**

**(A)** Sex prediction from methylation data accurately predicts participants reported sex (N=96).

**(B)** SNP-based hierarchical clustering.

**(C)** Quantile normalization robustly removes variation from raw data.

**(D)** Hierarchical clustering of all participants shows clustering by participant (N=96).

**(E)** Global PCA analysis of all beta-values neither shows significant separation when resolved by time (top) nor by diet (bottom) (N=96).

**(F)** Mean promotor methylation of all promotor-associated sites at baseline and after treatment in VD (left, N=48 samples, 24 paired measurements before-after) and MR (right, N=48 samples, 24 paired measurements before-after).

**Supplementary Figure 2:**


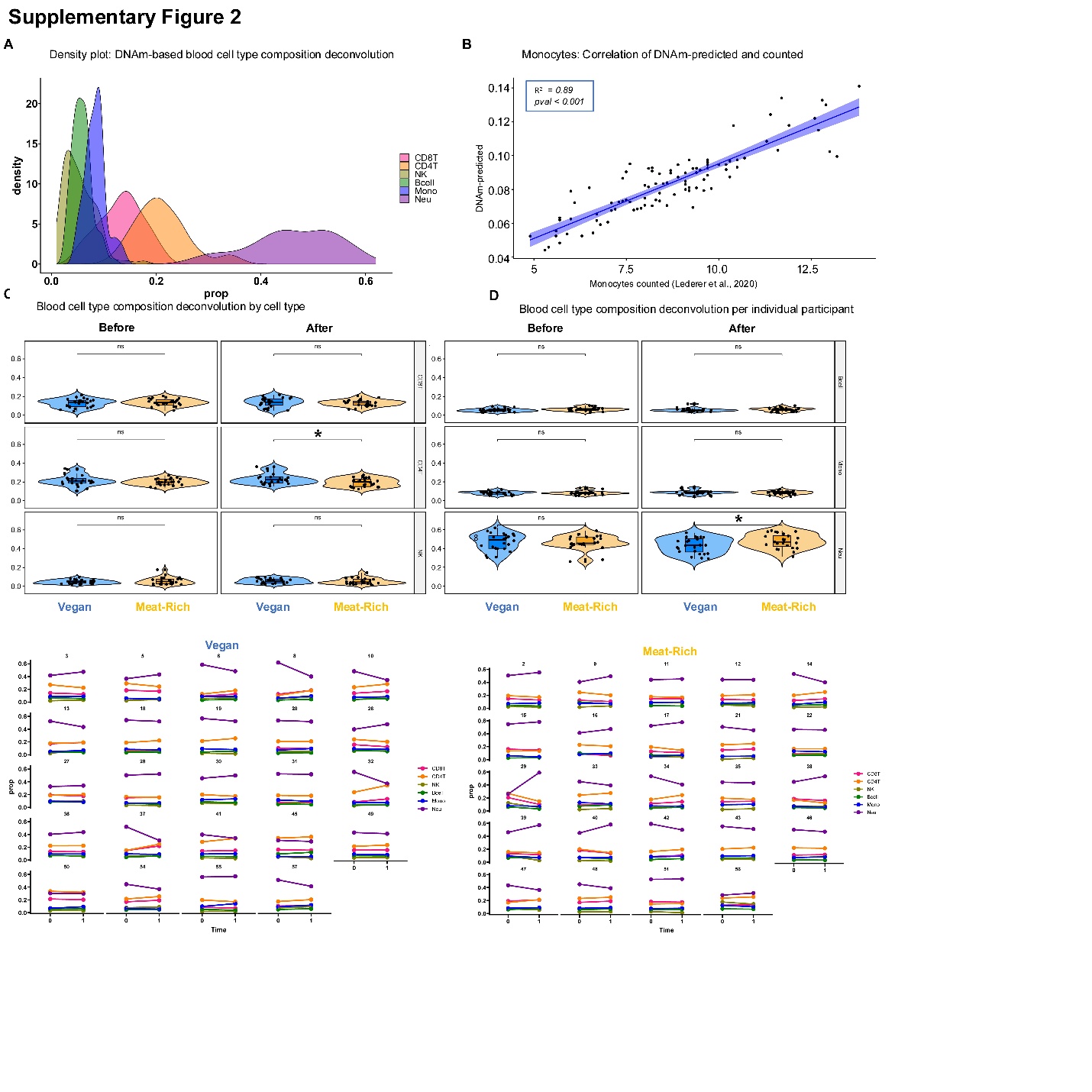


**Supplementary Figure 2 legend:**

**(A)** Predicted fractions of leukocyte cell types inferred from CpG methylation in all tested participants (N=96).

**(B)** Predicted monocyte fraction accurately matches monocyte counts from blood samples ([24]) by linear regression (R^2^=0.89, pval<0.001, N=96).

**(C-D)** Predicted changes in blood cell-type composition between MR and VD participants before and after dietary intervention as grouped comparison (C) and shown for each individual (D). CD4T-cell proportions decrease in MR after intervention, while Neutrophile proportions are elevated. (Welch’s two-sided t-test, *p<0.05, N=24). Differences in Neutrophile composition appeared to mainly be driven by a subset of participants, indicating the presence of responders and non-responders to the intervention.

**Supplementary Figure 3:**


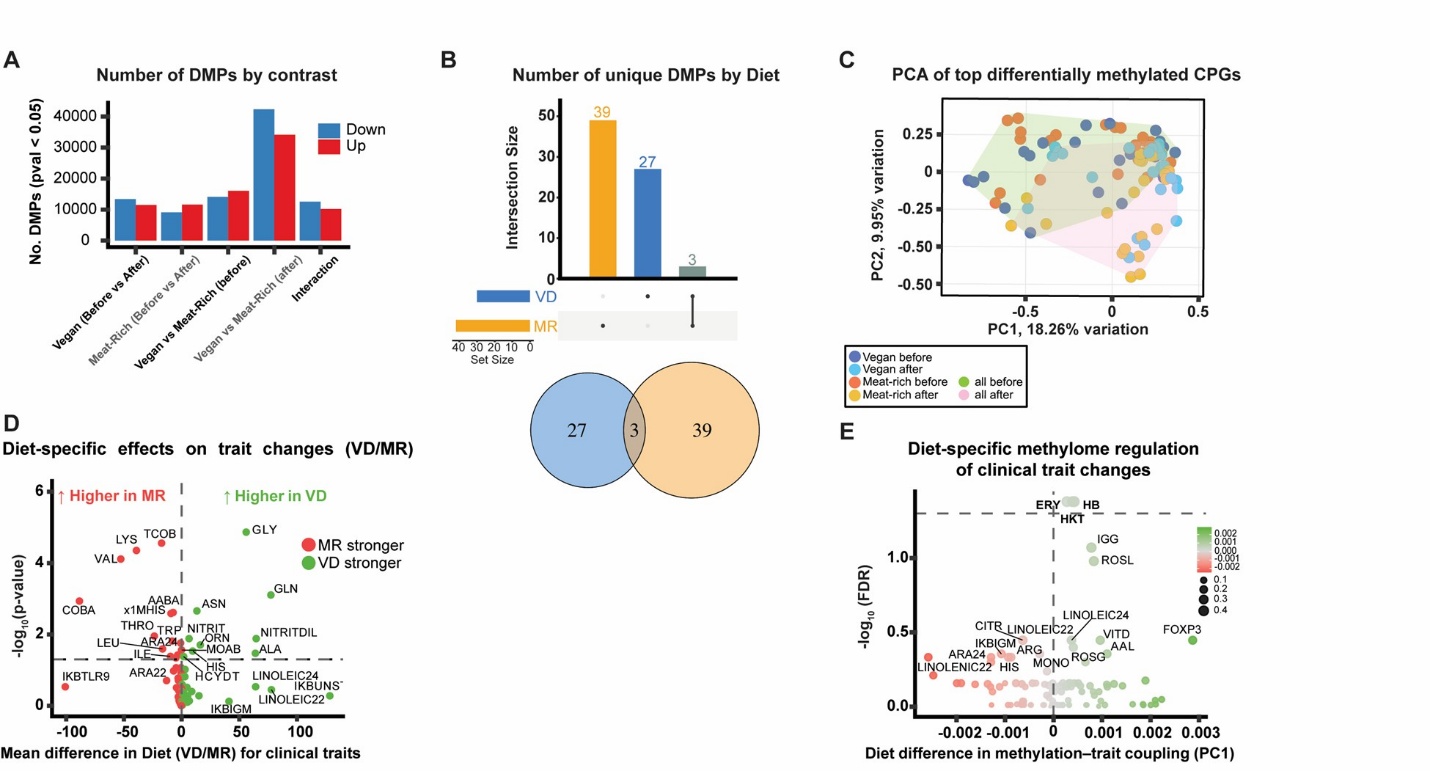


**Supplementary Figure 3 legend:**

**(A)** Bar plot illustrating the magnitude of all up- and down-regulated differentially methylated positions (DMPs), based on non-adjusted p-values (non-adjusted pvalue<0.05, |log2FC| >0).

**(B)** Intersections of significant DMPs (non-adjusted pvalue<0.05 and |log2FC|>0.5) found exclusively in either the vegan or meat-rich cohorts after the respective dietary intervention, and those shared between them.

**(C)** PCA of all DMPs found between VD and MR shows separation of samples between ‘before’ from ‘after’.

**(D)** Volcano plot showing diet-associated effects on changes in clinically measured traits post dietary Intervention.

**(E)** Volcano plot showing diet-dependent differences in the association of global methylation variation and changes in clinical traits, adjusted for sex and age. Values stronger for either diet, indicate a stronger methylation-trait relationship within that group.


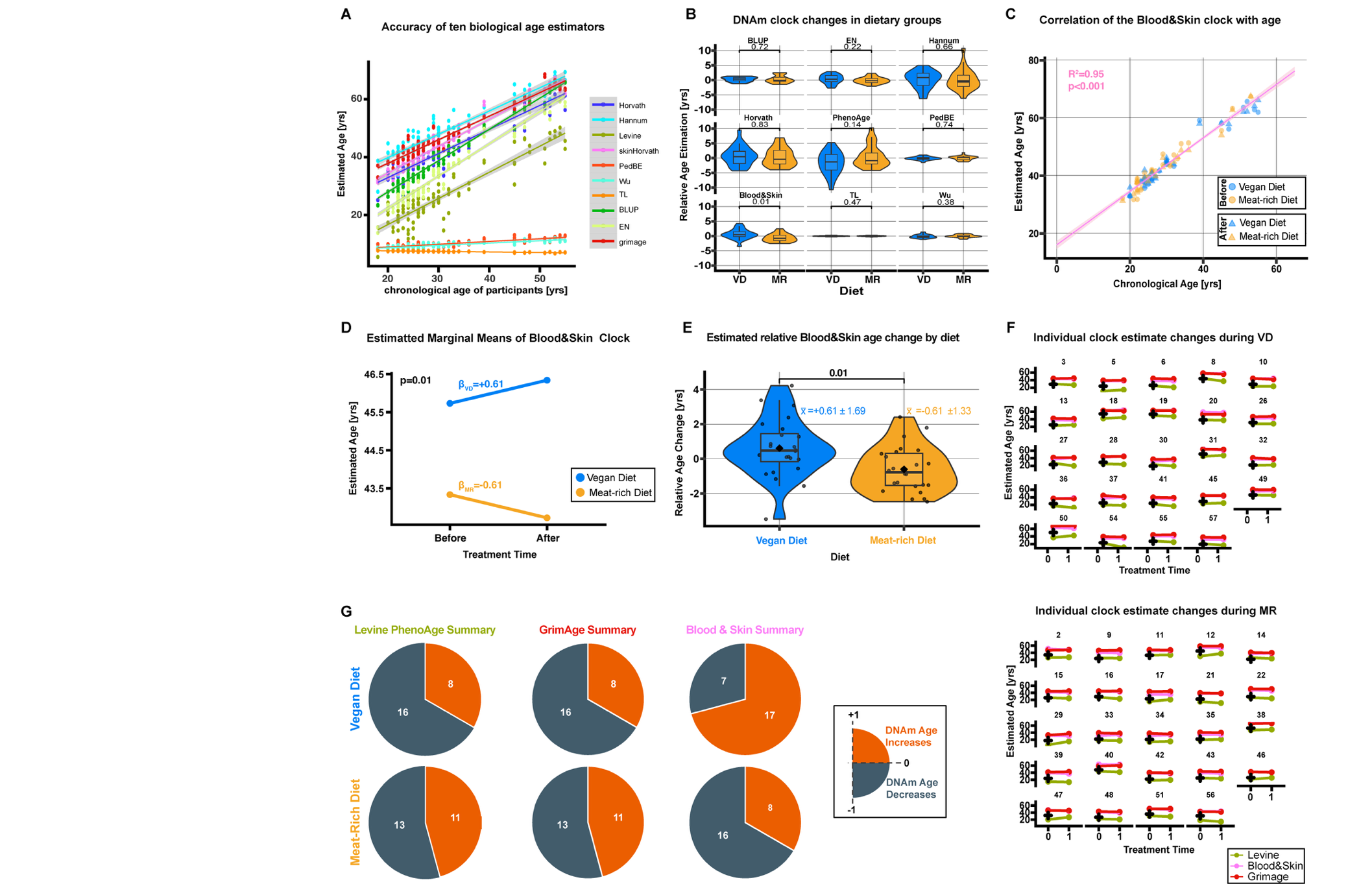


**Supplementary Figure 4:**

**Supplementary Figure 4 legend:**

**(A)** Comparison of ten different DNAm algorithms from participants blood sam+ples show various degrees of prediction accuracy in reference to the participants’ chronological age. Horvath’s Skin and Blood clock and Levine’s PhenoAge emerge as the most reliable models for age prediction from human blood samples (N=96).

**(B)** Baseline-normalization shows a significant decrease of estimated DNAm age with Horvarth’s Skin and Blood Clock (‘skinHorvarth’) in MR participants after dietary intervention, but not in other algorithms (VD: N=24, MR: N=24).

**(C)** The Horvath Skin and Blood clock shows a high correlation (R^2^=0.95, pvalue<0.001) between estimated methylation-based age and actual chronological age of all participants (N=96).

**(D)** Estimated marginal means of the Blood and Skin DNA methylation age prediction from a linear mixed effect model, using Diet x Time as fixed effects, and participant as a random effect (Diet x Time p=0.12), (VD: β=+0.61, 95% CI -0.02, +1.23], N=24; MR: β =-0.61, 95% CI [-1.23, +0.01], N=24).

**(E)** Baseline-normalized DNAm-scores predicted by the Blood & Skin clock shows a significant decrease in age of the MR cohort compared to VD (VD: N=24, MR: N=24). Points represent estimated relative age per donor; Diamond represents the mean value.

(F) Line plots showing the estimated age of the VD cohort (top) and MR cohort (bottom) from before to after treatment for each participant as predicted by the PhenoAge, GrimAge, and Blood & Skin estimators.

(G) Summary of F showing the amount of participants that either experience increase or decrease in estimated age during dietary intervention.
